# Protective factors and critical mucosal thickness for the stability of the peri‐implant soft tissue margin over 5 years: A longitudinal cohort study

**DOI:** 10.1002/jper.70159

**Published:** 2026-07-12

**Authors:** Lorenzo Tavelli, Hamoun Sabri, Erfan Barootchi, Martina Stefanini, Giovanni Zucchelli, Hom‐Lay Wang, Istvan A. Urban, Shayan Barootchi

**Affiliations:** ^1^ Department of Oral Medicine, Infection, and Immunity Division of Periodontology Harvard School of Dental Medicine Boston Massachusetts USA; ^2^ Department of Periodontics & Oral Medicine University of Michigan School of Dentistry Ann Arbor Michigan USA; ^3^ School of Dentistry Universidad Catolica de Guayaquil Guayaquil Ecuador; ^4^ Center for clinical research and evidence synthesis in oral tissue regeneration (CRITERION) Boston Massachusetts USA; ^5^ Emergency Medicine Department Tehran University of Medical Sciences Tehran Iran; ^6^ Department of Biomedical and Neuromotor Sciences University of Bologna Bologna Italy; ^7^ Urban Regeneration Institute Budapest Hungary

**Keywords:** buccal bone, implant complications, implant recession, mucosal recession, peri‐implant soft tissue dehiscences, phenotype, soft tissue stability

## Abstract

**Background:**

Peri‐implant soft tissue dehiscence deficiencies (PSTDs) are common esthetic complications in the anterior maxilla, yet the parameters that protect the peri‐implant soft tissue margin (STM) over time remain unclear. Previous studies have identified several risk indicators, but longitudinal thresholds capable of predicting stable STM have not been established. This study aimed to evaluate 5‐year STM behavior and identify clinically meaningful predictors of long‐term stability.

**Methods:**

This prospective longitudinal study included patients with anterior single implants with or without PSTD. Clinical and ultrasonographic examinations were performed at baseline (*T*_0_) and 5 years (*T*_5_), and included STM stability (primary outcome), PSTD incidence, mucosal thickness at 1 and 3 mm from the STM (MT1 and MT3), keratinized mucosa (KM) width, buccal bone thickness (BBT), and buccal bone dehiscence (BBD). Logistic regression, receiver operating characteristic curve (ROC) analysis, stratified subgroup analysis, and correlation testing were performed.

**Results:**

A total of 133 implants in 124 patients were evaluated. PSTD prevalence increased from 51.1% at *T*_0_ to 74.4% at *T*_5_, (incidence 47.7%). MT3 was the dominant predictor of STM stability (AUC = 0.998). The optimal statistical threshold was 2.01 mm, while a clinical stability threshold of 2.23 mm yielded 100% stability in this dataset. Other predictors (MT1, KM, BBT) were significant only in univariate models. In implants without PSTD at *T*_0_, MT3 alone predicted stability, whereas in implants with PSTD at *T*_0_, multiple variables influenced STM outcomes.

**Conclusions:**

MT3 is the critical determinant of 5‐year STM stability at anterior implants. In our cohort, achieving a MT3 ≥ 2.23 mm was found to be associated with a long‐term stable peri‐implant soft tissue.

## Plain language summary

Many people receive dental implants in the front part of the upper jaw, where appearance is very important. Over time, the gum line around an implant may move downward (away from the crown of the tooth), making the tooth look longer or even exposing metal parts. This is called a peri‐implant soft tissue deficiency (PSTD). Until now, it has not been clear which features of the gum and bone can best protect the gum line from changing over the years. In this study, patients with single implants in the esthetic zone were examined a first time and then also after 5 years. The researchers measured different tissue characteristics, including the thickness of the gum at 1 and 3 mm from the gum line, the width of the firm keratinized tissue, and the thickness and position of the bone in front of the implant. The goal was to find which of these features could reliably predict whether the gum line would stay stable over 5 years. A total of 133 implants were evaluated. More sites showed PSTD after 5 years than at the beginning of the study. Among all the measurements, the thickness of the gum 3 mm below the gum line (called MT3) was the most important predictor of long‐term stability. When MT3 was at least 2.23 mm, the gum line did not change over the 5 years in any of the patients. Other factors, such as bone or keratinized tissue width, were helpful but not as strong as MT3. These results show that ensuring adequate gum thickness around implants, especially at the deeper level, is critical for keeping the gum line stable over time and improving esthetic results.

## 1 INTRODUCTION

Dental implants are now a predictable option for replacing missing teeth,^1^, ^2^ and with their widespread use, the emphasis has shifted from survival to esthetic excellence, particularly in the anterior maxilla.^3^, ^4^ Among the most frequent esthetic complications is the apical migration of the peri‐implant soft tissue margin (STM), which can negatively affect patient satisfaction even when implants remain healthy.^5^, ^6^

Terminology describing these soft‐tissue deficiencies has historically been inconsistent, with terms such as “recession” and “dehiscence” used interchangeably.^7^, ^8^, ^9^ In 2019, Zucchelli et al. proposed a unified definition for the anterior maxilla: peri‐implant soft tissue dehiscence/deficiency (PSTD), characterized by an apical shift of the STM relative to the ideal position of the gingival margin of the homologous contralateral tooth.^5^ A recent systematic review by Tavelli and Barootchi identified 183 studies reporting soft‐tissue changes at implant sites and emphasized the need to differentiate PSTD from mucosal recession.^7^ Historically, mucosal recession referred exclusively to the exposure of the implant shoulder, abutment, or implant surface,^8^, ^10^ whereas PSTD is defined by the position of the STM relative to the contralateral tooth and is the preferred term for implants in the esthetic zone.^5^, ^7^, ^11^, ^12^, ^13^ This distinction is clinically important because an implant in the anterior maxilla may present with a long crown and an apically displaced STM—without exposing any implant components—yet still be perceived by patients as an esthetic failure.^6^, ^14^

Our group previously conducted a cross‐sectional study on healthy anterior implants and found that more than half of the patients and sites presented with PSTDs.^15^ Several factors were associated with these deficiencies, including limited mucosal thickness (MT), reduced keratinized mucosa (KM) width, and buccal bone dehiscence (BBD), among others.^15^ Although cross‐sectional studies help identify risk indicators, longitudinal data are needed to determine prognostic thresholds and clarify which parameters genuinely protect the peri‐implant STM over time.

For this reason, the present study was designed to evaluate the behavior of the STM over 5 years and to establish clinically meaningful protective factors for maintaining stable soft tissue levels at anterior implant sites.

## 2 MATERIALS AND METHODS

### 2.1 Study registration and design

The current study was approved by the University of Michigan Medical School Institutional Review Board (IRBMED) (HUM00176741), in accordance with the Helsinki Declaration of 1975, as revised in 2013. An informed consent was obtained from all individuals who had participated in the study. The present manuscript follows the Strengthening the Reporting of Observational Studies in Epidemiology (STROBE) statement for improving the quality of reports of observational studies.^16^

### 2.2 Participants and clinical and ultrasonographic measurements

Subjects with one or more clinically healthy dental implants ^17^ in the esthetic area (from the right first premolar to the left first premolar) attending the Graduate Periodontics clinic at the Department of Periodontics, University of Michigan, Ann Arbor, were identified and recruited as described in the original publication ^15^ and in the Supplementary Appendix.

Based on the presence or absence of PSTD at baseline, subjects were allocated into two groups: implants without PSTD at *T*_0_ (non‐PSTD group) and implants with PSTD at *T*_0_ (PSTD group).

At each study visit (baseline [*T*_0_] and at the 5‐year follow‐up [*T*_5_]), patient demographics, implant characteristics, and adverse events at the study site during the last 5 years (e.g., changes in implant health status) were recorded. Implants that received any soft tissue augmentation or reconstructive surgical procedure at the study site between *T*_0_ and *T*_5_ were excluded from the analysis, in order to preserve the natural history of the peri‐implant STM. In addition, clinical measures of interest, including the presence or absence of PSTD,^5^ probing depth, and the KM width at the midfacial aspect were assessed (Supplementary Appendix). The clinical examinations were conducted two calibrated clinicians (S.B. and L.T.), with intra‐ and inter‐examiner reliability confirmed prior to data collection. PSTD change between *T*_0_ and *T*_5_ was assessed as the apico‐coronal shift of the peri‐implant STM through superimposing digital impressions.^18^, ^19^

The ultrasound equipment setup and the scanning procedures have been previously described in detail.^20^, ^21^, ^22^, ^23^, ^24^ By orienting the ultrasound probe perpendicular to the occlusal plane and parallel to the long axis of the implant at its midfacial aspect, ultrasound scans were obtained and analyzed to assess MT, that was evaluated at 1 and 3 mm from the STM (MT1 and MT3, respectively), BBD, and peri‐implant buccal bone thickness (BBT). Additional information are reported in the Supplementary Appendix.

In addition, at *T*_5_, subjects were asked to rate esthetics (EST) at each implant site using a 100 mm visual analogue scale (VAS) and to indicate the willingness to undergo treatment.

Patients were followed under a standardized supportive periodontal and peri‐implant care protocol consisting of professional prophylaxis and clinical re‐evaluation every 3–4 months for patients with history of periodontal disease, and every 6 months for patients without history of periodontal disease.

### 2.3 Study outcomes

The primary outcome of the study was the behavior of the STM over 5 years, and the identification of baseline predictors associated with long‐term STM stability. In this study, STM stability refers to the absence of apical shift of the STM over the observation period, regardless of whether a PSTD was present at baseline. A PSTD may be present from the moment of crown delivery due to a long crown or suboptimal implant positioning, without any prior tissue migration having occurred; therefore, stability of the STM represents a genuinely positive biological outcome in both groups. Secondary outcomes included the assessment of the incidence of PSTD over this time period, the identification of a critical threshold for the most determinant parameters at *T*_0_ able to predict long‐term stability, and the evaluation of the impact of PSTD on patient‐reported esthetics and willingness to pursue treatment

### 2.4 Statistical analyses

The implant was the unit of analysis. Descriptive statistics were calculated for all baseline and 5‐year parameters, with continuous variables reported as mean ± standard deviation and categorical variables as frequencies and percentages.

#### 2.4.1 Predictor analysis

To identify baseline (*T*_0_) parameters associated with the 5‐year stability of the peri‐implant STM, univariate analysis was conducted. Simple logistic regression was used for continuous variables (e.g., MT1, MT3, KM width) to calculate odds ratios (OR) and 95% confidence intervals (CI). Chi‐squared or Fisher's exact tests were employed for categorical variables.

#### 2.4.2 Critical threshold identification

Receiver operating characteristic (ROC) curve analysis was performed for the most determinant baseline parameters to assess predictive power and identify optimal clinical thresholds. The area under the curve (AUC) quantified discriminatory ability, and the optimal statistical cutoff was identified using the Youden Index. A clinical stability threshold was determined as the minimum value above which 100% of implants maintained STM stability.

#### 2.4.3 Advanced statistical analyses

A multi‐level logistic regression model was planned to determine independent predictor contributions.

Additional analyses included linear regression for tissue change magnitude, interaction analysis for synergistic effects (MT3 × KT, MT3 × BBT, MT3 × BBD), Pearson correlation analysis among baseline anatomical variables, subgroup analysis by initial PSTD status, esthetic outcomes assessment, and longitudinal comparisons using paired and independent non‐parametric tests.

Detailed statistical methods are provided in detail in the Supplementary Appendix.

## 3 RESULTS

### 3.1 Study population and PSTD outcomes

A total of 133 implants in 124 patients were included in the 5‐year analysis. At baseline, 65 implants were classified as non‐PSTD and 68 as PSTD. At *T*_5_, the overall prevalence of PSTD increased from 51.1% to 74.4%; the 5‐year incidence of new PSTD cases was 47.7% (31 out of 65 initially non‐PSTD implants), while all 68 PSTD implants at baseline remained affected at 5 years.

The changes in clinical and ultrasonographic outcomes between *T*_0_ and *T*_5_ are reported in Table 1. Limited and not significant changes were observed for the majority of the outcomes (PSTD depth, probing depth, KM width, MT1, MT3, and BBT), while a substantial increase in BBD was found within the two groups (on average, BBD increase of 1.20 mm and 0.92 mm in the non‐PSTD group and in the PSTD group, respectively).

**TABLE 1:** Comparison of clinical and ultrasonographic outcomes between non‐PSTD and PSTD implants, as classified at baseline.

Variable	Time	Non‐PSTD group (*n* = 65) Mean ± SD	PSTD group (*n* = 68) Mean ± SD	Between‐group *p*‐value
PSTD depth (mm)	*T*_0_	0.00 ± 0.00	2.25 ± 3.63	<0.001*
	*T*_5_	0.44 ± 0.49	2.46 ± 1.02	<0.001*
	Change	0.44	0.21	
Probing depth (mm)	*T*_0_	2.62 ± 0.84	2.62 ± 0.61	0.528
	*T*_5_	2.66 ± 0.82	2.66 ± 0.62	0.607
	Change	0.04	0.04	
KM width (mm)	*T*_0_	4.48 ± 1.70	2.21 ± 1.70	<0.001*
	*T*_5_	4.52 ± 1.67	2.32 ± 1.69	<0.001*
	Change	0.04	0.11	
MT1 (mm)	*T*_0_	1.43 ± 0.57	0.95 ± 0.38	<0.001*
	*T*_5_	1.37 ± 0.61	0.96 ± 0.38	<0.001*
	Change	−0.06	0.01	
MT3 (mm)	*T*_0_	2.04 ± 0.83	1.61 ± 0.54	0.001*
	*T*_5_	1.95 ± 0.83	1.56 ± 0.53	0.002*
	Change	−0.09	−0.05	
BBD (mm)	*T*_0_	1.83 ± 1.23	3.55 ± 2.17	<0.001*
	*T*_5_	3.03 ± 1.75	4.46 ± 2.11	<0.001*
	Change	1.20	0.92	
BBT (mm)	*T*_0_	1.50 ± 0.71	0.93 ± 0.42	<0.001†††
	*T*_5_	1.71 ± 0.78	1.11 ± 0.57	<0.001†††
	Change	0.22	0.18	

*Note*: The Mann–Whitney U test was used for between‐group comparisons.

Abbreviations: BBD, buccal bone dehiscence; BBT, buccal bone thickness; KM, keratinized mucosa; MT1, mucosal thickness assessed 1 mm apical to the soft tissue margin; MT3, mucosal thickness assessed 3 mm apical to the soft tissue margin; PSTD, peri‐implant soft tissue dehiscence; SD, standard deviation; T0, baseline; T5, 5‐year follow‐up.

*Denotes statistical significance.

### 3.2 Univariate analysis of baseline predictors for stability

The univariate logistic regression analysis identified several baseline parameters as significant predictors of STM stability over 5 years. The results are summarized in Table 2.

**TABLE 2:** Univariate logistic regression assessing the baseline predictors of STM stability at 5 years.

Variable (at baseline)	Odds ratio	95% CI	*p*‐value
MT3 (mm)	Very High^†^	—	0.006*
MT1 (mm)	4.27	2.02–9.01	0.0001*
KM width (mm)	1.29	1.08–1.55	0.006*
BBT (mm)	1.99	1.12–3.54	0.019*
BBD (mm)	0.90	0.74–1.10	0.308
PSTD depth (mm)	0.44	0.28–0.69	0.0003*
Years in function of the implant	0.94	0.85–1.03	0.193
Presence of adjacent implant (y/n) ^‡^	—	—	0.576

*Note*: A multivariate logistic regression model could not be fitted due to quasi‐complete separation driven by MT3, which near‐perfectly predicted the outcome; therefore, only univariate estimates are reported. A multivariate linear regression model using PSTD depth change as a continuous outcome is presented in Table S3.

Abbreviations: BBD, buccal bone dehiscence; BBT, buccal bone thickness; CI, confidence interval; KM, keratinized mucosa; MT1, mucosal thickness assessed 1 mm apical to the soft tissue margin; MT3, mucosal thickness assessed 3 mm apical to the soft tissue margin; PSTD, peri‐implant soft tissue dehiscence; STM, soft tissue margin.

*Denotes statistical significance.

†The odds ratio for MT3 was extremely high, indicating quasi‐complete separation and confirming its dominant predictive power.

‡ Categorical variables analyzed with chi‐squared/Fisher's exact tests; odds ratio not applicable.

Thicker baseline mucosal tissue (MT3 and MT1), wider KM, and greater BBT were identified as significant positive predictors of STM stability. Conversely, a greater PSTD depth at baseline was a significant negative predictor.

### 3.3 Critical threshold analysis

Given its significance in the univariate analysis, ROC curve analysis was performed to determine the predictive power of baseline MT3 for 5‐year stability of the STM. The analysis yielded an AUC of 0.998.

Further analysis revealed two important and distinct thresholds:

1. The Statistically Optimal Threshold (2.01 mm): The Youden Index identified 2.01 mm as the cutoff that mathematically maximizes the combination of sensitivity (100%) and specificity (96.6%). The clear separation in MT3 values between the stable and not stable STM groups, with the 2.01 mm threshold, is visualized in Figure 1.
2. The Clinical Stability Threshold (2.23 mm): A detailed examination of the data revealed that while some implants with MT3 between 2.01 mm and 2.23 mm showed an apical migration of the STM, 100% of implants (*n* = 37) with a baseline MT3 of ≥ 2.23 mm showed a stable STM over 5 years. This provides a definitive “safe zone” for clinical decision‐making. This dual‐threshold model allows for a more nuanced clinical interpretation, defining three distinct zones of risk for apical shift of the STM (Table S1). The stark difference in outcomes between these three zones is visualized in Figure 2. The log‐rank tests comparing the outcomes for these groups were all highly significant (*p* < 0.001), confirming they represent statistically distinct populations. It should be noted that this threshold was derived from the overall cohort. The stratified analysis by baseline PSTD status, presented in Section 3.6, shows that the predictive role of MT3 applies to both groups, although in implants with PSTD at baseline, a multitude of additional factors also contribute to long‐term STM stability.

**FIGURE 1:**
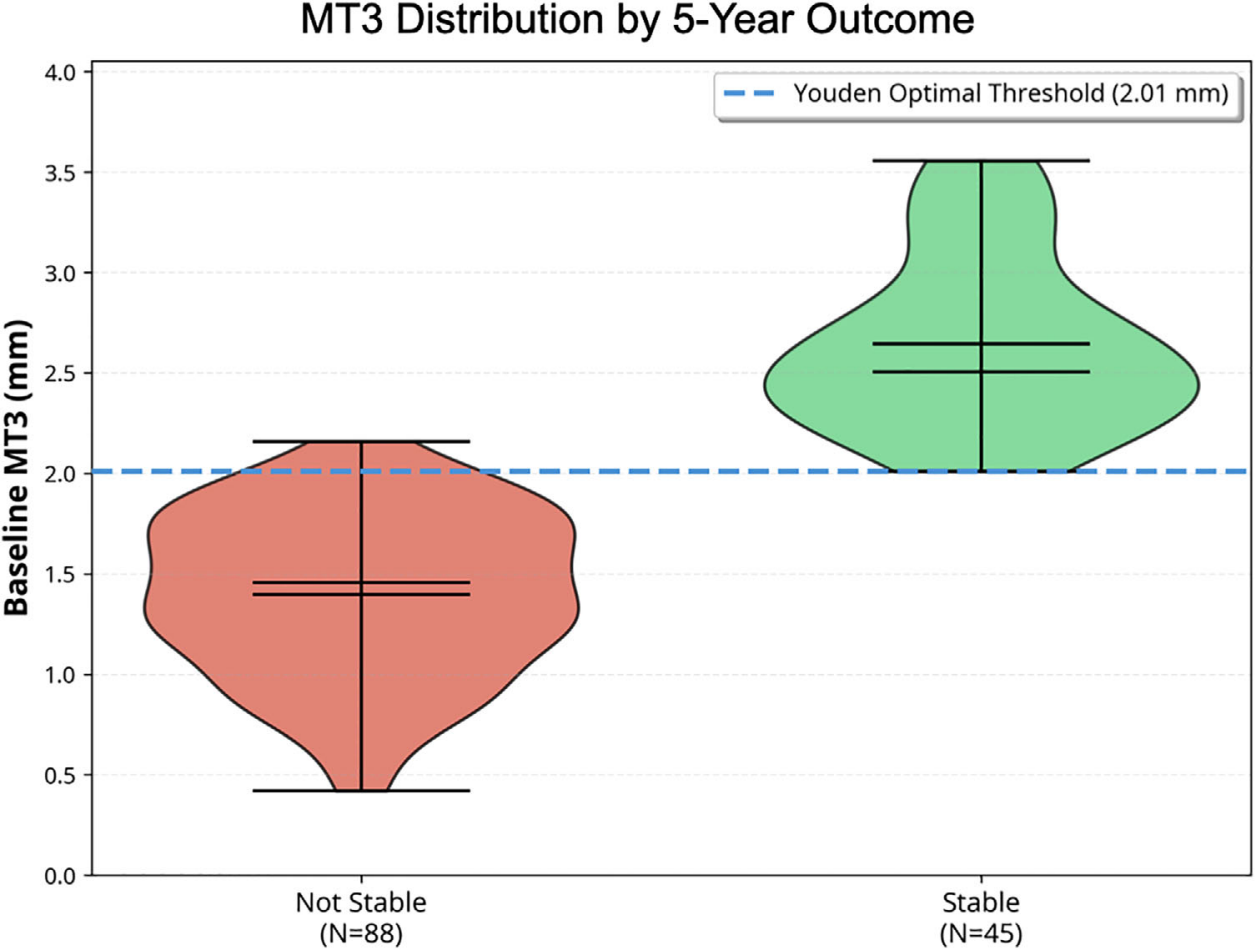
Violin plot distribution of baseline MT3 values by 5‐year outcome. MT3, mucosal thickness assessed 3 mm apical to the soft tissue margin.

**FIGURE 2:**
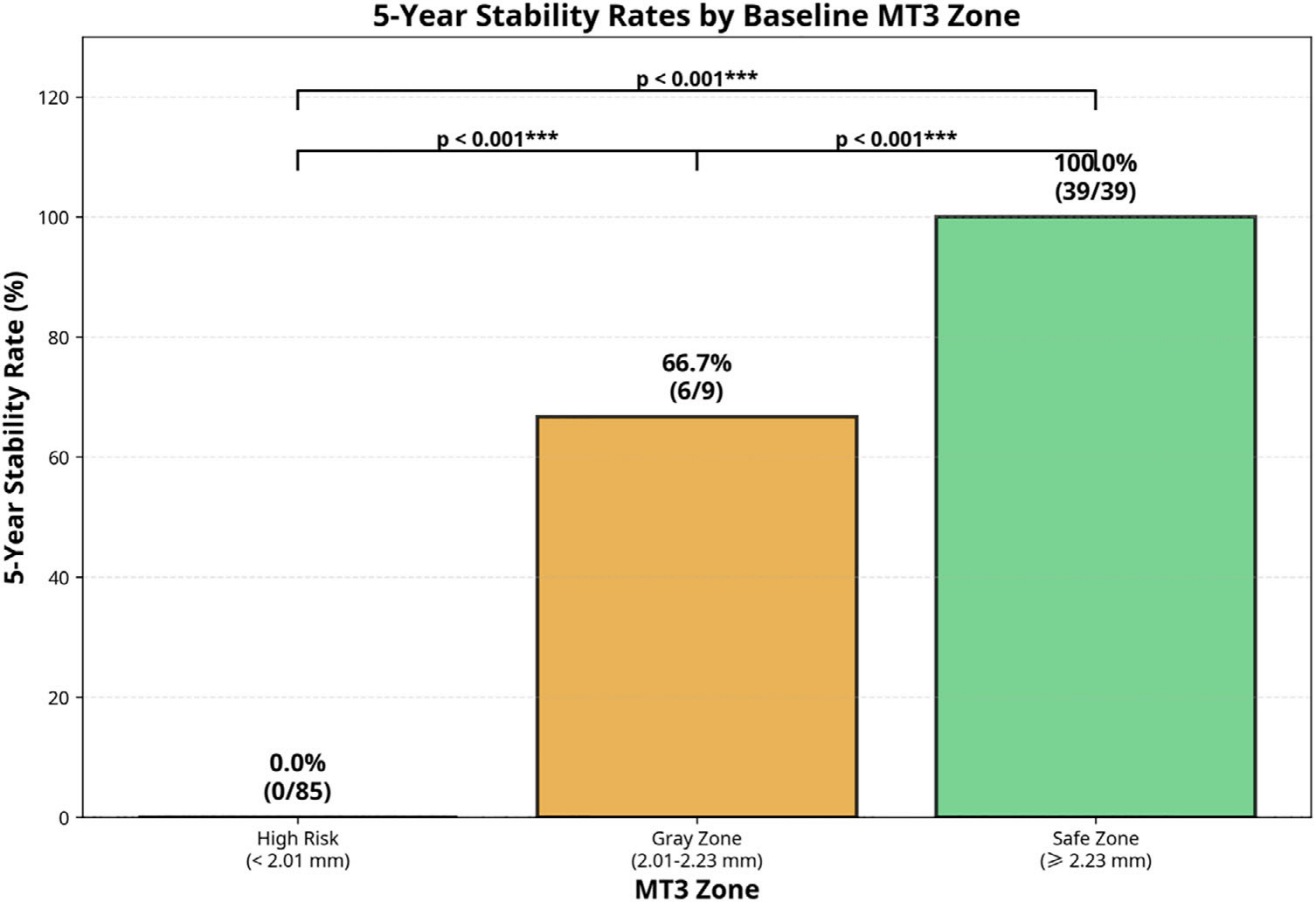
Five‐year STM stability rates by baseline MT3 zone, with statistical significance brackets (p < 0.001 for all comparisons). MT3, mucosal thickness assessed 3 mm apical to the soft tissue margin; STM, soft tissue margin.

### 3.4 Multi‐level analyses

A multi‐level logistic regression model was attempted to evaluate the independent contribution of the significant predictors identified in the univariate analysis, in terms of STM stability. However, the model failed to converge because of quasi‐complete separation. This occurs when one variable (in this case baseline MT3) almost perfectly predicts the primary outcome, preventing the computation of stable coefficients for the remaining variables.

To further quantify the impact of baseline predictors on the magnitude of STM changes, a linear regression analysis was performed using the 5‐year change in PSTD depth as the outcome variable. In the univariate analysis, baseline MT3 was again the most powerful predictor, explaining 65.2% of the variance (*R* ^2^ = 0.652, *p* < 0.0001) in PSTD depth change. For every 1 mm increase in baseline MT3, the apical shift of the STM was reduced by 0.51 mm. While baseline MT1 was also significant (*p* = 0.002), no other predictors showed a significant relationship with the magnitude of change (Table S2). In a multivariate model including both MT3 and MT1, MT3 remained the sole significant predictor (*p* < 0.0001), while MT1 became non‐significant (*p* = 0.381) (Table S3). This confirms that the protective effect of mucosal thickness is primarily driven by its dimension at the 3 mm level, which single‐handedly accounts for over 65% of the long‐term dimensional changes of the peri‐implant STM (Figure 3A).

**FIGURE 3:**
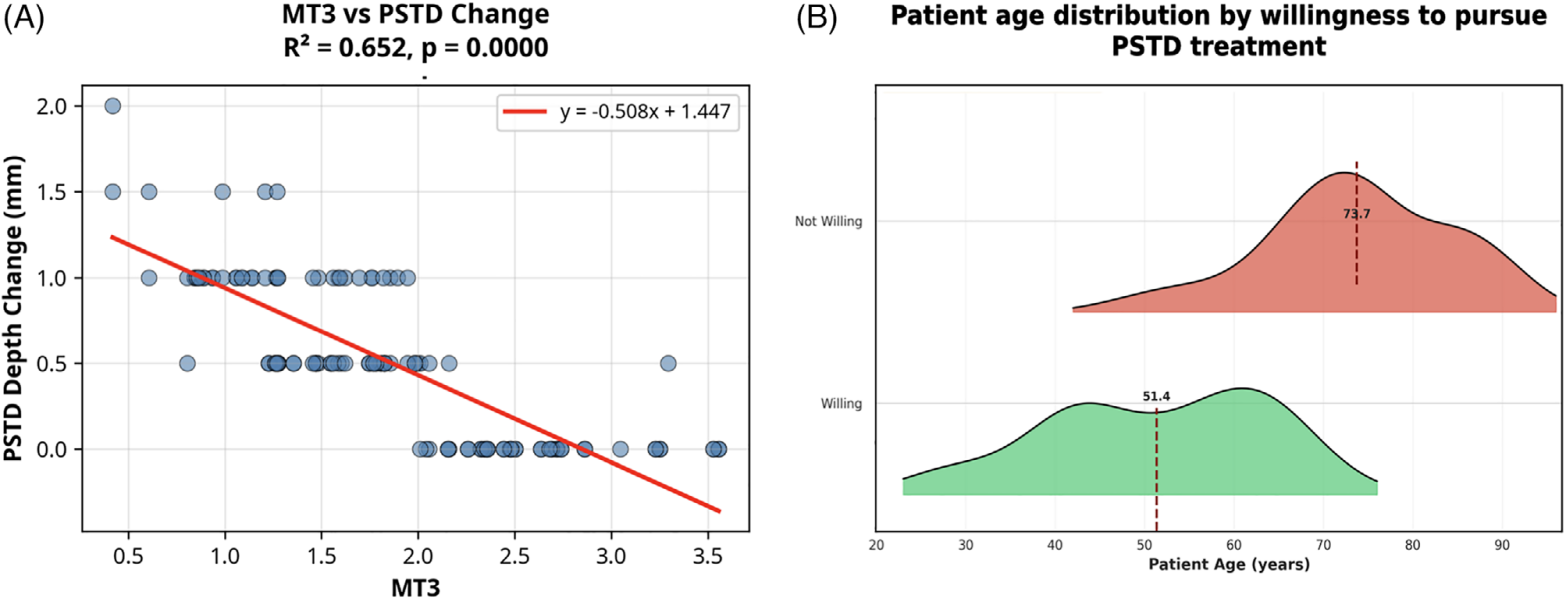
(A) Linear regression plot showing the strong negative correlation between baseline MT3 and the 5‐year change in PSTD depth. MT3 alone explains 65.2% of the variance in the outcome. (B) Ridge plot showing patient age distribution for willing (green) versus not willing (red) to pursue PSTD treatment. Dashed lines indicate mean values. Mann–Whitney U test, *p* < 0.0001. MT3, mucosal thickness assessed 3 mm apical to the soft tissue margin; PSTD, peri‐implant soft tissue dehiscence/deficiency.

### 3.5 Inter‐relationship of predictors

To explore the relationships between the key anatomical variables, a Pearson correlation matrix was generated. The analysis revealed only weak to moderate correlations among the variables of interest, indicating that they represent distinct anatomical features rather than being proxies for one another. The strongest association was a moderate positive correlation between KM width and BBT (r = 0.523, *p* < 0.0001), suggesting a pattern consistent with a generally thicker tissue phenotype. The other significant correlations were weak to weak‐moderate, indicating that the predictors represent distinct anatomical features rather than being proxies for one another. The negative, weak‐moderate correlations with BBD suggest that thinner tissue (MT1, KM width, and BBT) tend to be associated with a greater extent of BBD (Table S4 and Figure S1).

Furthermore, to investigate potential synergistic effects, interaction analyses (MT3 × KT, MT3 × BBT, and MT3 × BBD) were performed. No statistically significant interactions were found. The lack of synergistic or antagonistic effects reinforces the finding that the predictors act independently and that no combination of other factors can compensate for a deficient MT3.

### 3.6 Subgroup analysis based on the initial PSTD status

To determine if the predictors of stability differed based on the initial condition of the soft tissue, a subgroup analysis was performed on implants that started with PSTD (*n* = 68) versus those that started without (non‐PSTD, *n* = 65).

- In the non‐PSTD group, where the outcome was true STM stability versus the incidence of new PSTD, baseline MT3 was the only significant continuous predictor of stability (2.76 mm for stable vs. 1.39 mm for unstable, *p* < 0.0001).
- In the PSTD group, where the outcome was the stability of the STM or its apical migration over 5 years, MT3 remained the most powerful predictor (2.39 mm vs. 1.40 mm, *p* < 0.0001). However, in this subgroup, MT1 (*p* = 0.005), BBT (*p* = 0.029), baseline PSTD depth (*p* = 0.016), and metal exposure (*p* = 0.0002) were also significant. This indicates that once PSTD is established, while MT is still primary, a multitude of other factors may contribute to the prognosis (Figure 4 and Tables S5).

**FIGURE 4:**
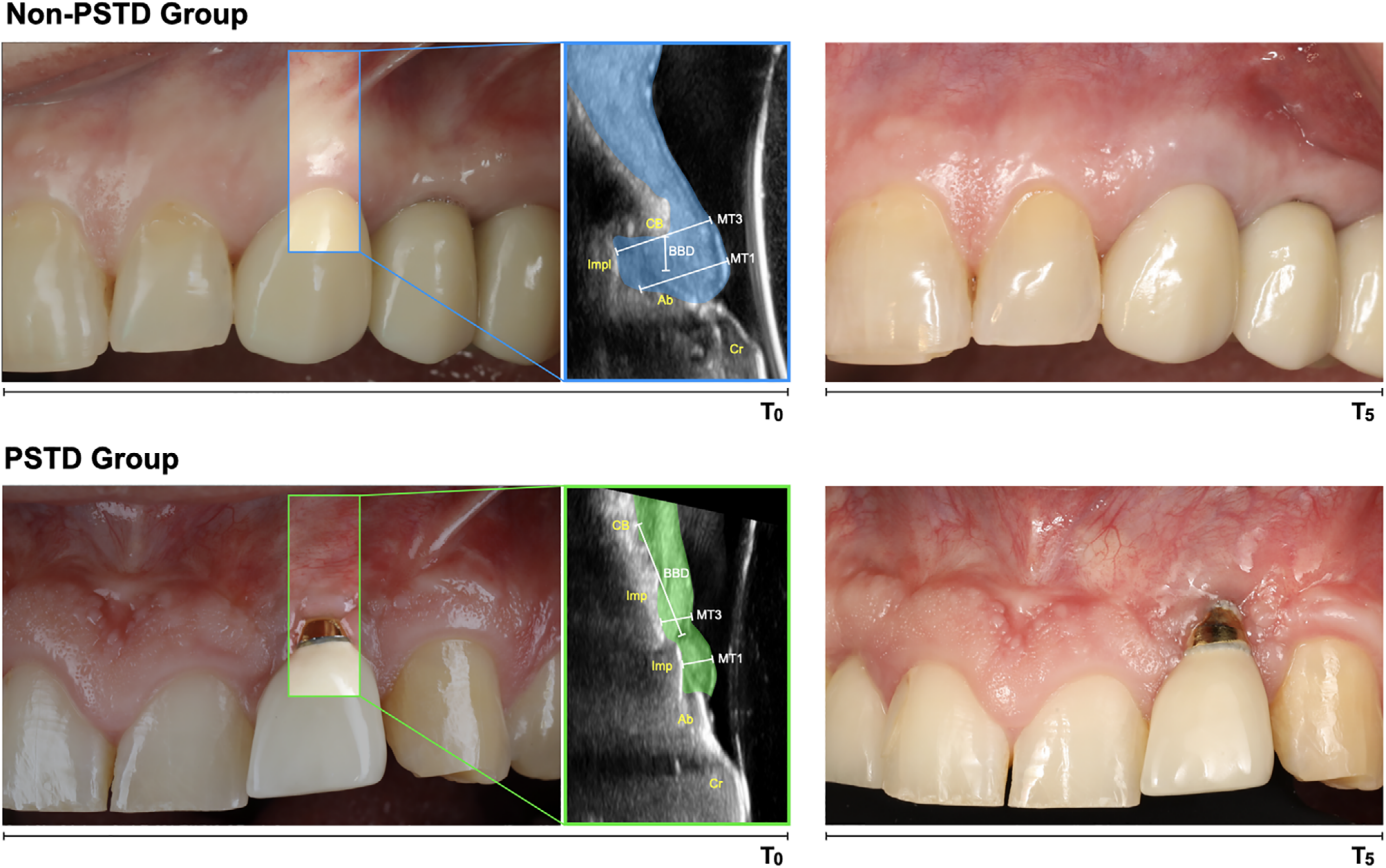
Example cases from the non‐PSTD and the PSTD groups. The upper panel shows a canine implant diagnosed without PSTD at T_0_. The ultrasonographic assessment is shown on the right side of the photograph, with the soft tissue compartment highlighted in blue. “Cr” identifies the implant‐supported crown, “Ab” the abutment, “Impl” the implant fixture, and “CB” the crestal bone. The values of MT1, MT3, BBD, and BBT were 2.78 mm, 2.92 mm, 0.85 mm, and 1.61 mm, respectively. The implant showed stable STM at T_5_, and remained classified as a non‐PSTD case. The lower panel presents an implant diagnosed with a PSTD at T_0_, characterized by a long implant‐supported crown and exposure of the abutment. The corresponding ultrasonographic scan is shown on the right of the baseline photograph, with the soft tissue compartment highlighted in green. The values of MT1, MT3, BBD, and BBT were 1.12 mm, 1.07 mm, 5.04 mm, and 0.93 mm, respectively. An apical shift of the STM of 1.5 mm was observed at T_5_, likely reflecting the suboptimal baseline value of several predictors of STM instability. BBD, buccal bone dehiscence; BBT, buccal bone thickness; MT1, mucosal thickness assessed 1 mm apical to the soft tissue margin; MT3, mucosal thickness assessed 3 mm apical to the soft tissue margin; PSTD, peri‐implant soft tissue dehiscence/deficiency; STM, soft tissue margin.

### 3.7 Patient‐reported outcome measures (PROMs)

Subjects rated EST significantly higher at sites without PSTD compared to sites exhibiting PSTD (95.2 ± 13.9 vs 56.4 ± 31.5 VAS, *p* < 0.001, respectively). EST was significantly associated to PSTD depth (*p* < 0.001) and exposure of metal components (26.5 vs 88.2 VAS, *p* < 0.001, at sites exposing vs sites not exposing metal components, respectively). Among the patients with PSTD at 5 years, only 39.4% expressed a willingness to pursue surgical treatment. Patient age was the only significant predictor of willingness to treat the condition (*p* < 0.001), with patients willing to pursue treatment being significantly younger compared to those not willing (mean age 51.4 ± 12.0 vs 73.7 ± 10.3 years, respectively, Figure 3B).

## 4 DISCUSSION

Previous literature has extensively examined how hard and soft tissue phenotype components affect peri‐implant outcomes.^7^, ^8^, ^10^, ^14^, ^25^, ^26^, ^27^, ^28^, ^29^, ^30^ Spray and colleagues were among the first to show that residual BBT at implant placement influences vertical bone levels at abutment connection, and suggested that a facial bone thickness of roughly 1.8 to 2.0 mm at placement is desirable to maintain a stable buccal bone crest.^26^ In a different context, Chappuis and coworkers identified a critical facial bone wall thickness around 1 mm in extraction sockets, with sites thinner than this threshold exhibiting pronounced vertical bone resorption after tooth loss.^31^ Other authors have proposed that at least 1.5 to 2.0 mm of BBT should be present to minimize physiologic and pathologic bone remodeling, especially in the esthetic zone.^25^, ^32^, ^33^ Together, these studies established the concept of a critical BBT as a key determinant of peri‐implant hard tissue stability.^25^, ^32^, ^33^

The role of peri‐implant KM has been explored more recently than that of BBT. While some early studies questioned the clinical relevance of KM width,^34^, ^35^ the majority of contemporary evidence indicates that an adequate band of firm and adherent KM is associated with less plaque accumulation, less soft tissue inflammation, less mucosal recession, and improved patient comfort.^36^, ^37^, ^38^, ^39^, ^40^, ^41^ Nonetheless, the exact threshold remains debated.^27^, ^29^, ^42^, ^43^ The often cited value of 2 mm may underestimate the true requirement in many clinical scenarios, and recent work on the peri‐implant phenotype has argued for a more nuanced and site specific interpretation rather than a single universal cutoff.^27^, ^29^, ^42^, ^43^

With regard to critical soft tissue thickness at implant sites, a series of investigations by Linkevicius, Puisys, and coworkers focused on the vertical component of the peri‐implant soft tissue, that is the supracrestal tissue height (STH).^44^, ^45^, ^46^ These studies consistently showed that a minimum STH is required to limit marginal bone remodeling.^44^, ^45^, ^46^ Longer term data suggested that a STH of approximately 3 mm around implants is associated with stable crestal bone levels over time, whereas thinner vertical tissues are linked to more pronounced bone level changes.^47^ This work introduced the idea that a critical STH can modulate the tolerance of the bone crest to functional loading and biologic remodeling.

The horizontal component of peri‐implant soft tissue thickness (MT) has historically been studied mainly for its impact on color and esthetic integration.^48^, ^49^, ^50^, ^51^, ^52^ Several clinical and experimental studies suggested that at least 2 mm of MT are needed to mask underlying metallic components and prevent soft tissue discoloration.^48^, ^49^, ^50^, ^51^, ^52^ More recently, attention has shifted to the possible influence of MT on peri implant health.^28^, ^53^, ^54^, ^55^ Multiple investigations have reported that implants with limited MT show greater marginal bone loss than implants with thicker or augmented soft tissues,^28^, ^53^, ^54^, ^55^ and one study proposed a cutoff around 2.88 mm of MT to preserve marginal bone levels.^56^ However, despite these contributions, no previous study has specifically evaluated the long‐term stability of the STM as the primary outcome, nor has any work identified a critical value of MT associated with stable STM over an extended period.

The present longitudinal study addresses this gap by demonstrating that MT measured at 3 mm apical to the STM (MT3) is the dominant predictor of 5‐year STM stability at anterior implants. Other parameters, including baseline MT1, KM width, BBT, and PSTD depth, still influence the overall behavior of the STM, but their contribution becomes secondary once MT3 is taken into account. Baseline MT3 showed near perfect predictive ability for STM stability, and ROC analysis identified a statistically optimal threshold of 2.01 mm that maximized sensitivity and specificity. A slightly higher clinical stability threshold of 2.23 mm emerged as the value above which all implants in this cohort maintained a stable STM over 5 years, defining a clear safe zone and three clinically distinct risk categories. These findings extend the existing evidence, at least for anterior implants already in function, by shifting the focus from bone‐centered end points to a truly soft tissue‐centered outcome.^7^, ^27^, ^57^, ^58^, ^59^, ^60^ It should be noted that this threshold reflects an empirical observation within this cohort and does not represent an absolute biological rule; implants with MT3 below 2.23 mm are not inevitably destined to develop or experience progression of PSTD but carry a higher risk of STM apical shift and warrant closer monitoring and individualized clinical management.

The quasi‐complete separation observed in the logistic model reinforces the strength of this association. Once MT3 reached the clinical stability threshold, apical migration of the STM became extremely unlikely. In contrast, implants with thinner tissues remained at risk despite similar values of KM width or BBT. This is consistent with a biologic rationale in which a thicker supracrestal soft tissue compartment can accommodate bone remodeling and functional loading without translating subtle changes at the bone crest into visible apical displacement of the STM.^3^, ^27^, ^29^, ^42^ It is noteworthy that previous suggestions of a minimum MT of about 2 mm for esthetic reasons ^50^, ^52^, ^61^ are remarkably consistent with the statistically optimal and clinically stable thresholds identified here for long‐term STM stability.

The hierarchical structure of protective factors is a key conceptual point. KM width was a significant predictor in the univariate analysis, but its effect lost statistical significance once MT3 was included in the model. This pattern suggests that the apparent protective effect of KM is largely mediated by the underlying soft tissue volume rather than representing an independent driver of stability. The moderate correlation observed between KM width and BBT supports the idea that a wider band of KM may be a marker of a generally thick tissue phenotype rather than a stand‐alone protective factor. Clinically, this implies that, at anterior implant sites, obtaining an adequate band of KM remains important, but it should not take precedence over ensuring sufficient MT.

A similar hierarchy emerged with respect to the hard tissue phenotype. BBT was a significant predictor in univariate analysis, but its influence became secondary once MT3 was considered. This does not diminish the relevance of adequate bone support for long‐term implant success. Rather, it underscores that the soft tissue serves as the primary protective layer and that bone thickness alone cannot prevent soft tissue recession if the overlying mucosa is deficient. The peri‐implant soft tissue acts as the first line of defense against mechanical and microbial challenges, and its adequacy seems to be a prerequisite for preserving both the hard tissue architecture and the level of the STM.^27^, ^42^

By contrast, the position of the buccal bone (BBD) did not emerge as a significant predictor of 5‐year STM stability in this cohort, which differs from some previous reports that linked more apical buccal bone to a higher risk of soft tissue complications.^10^, ^62^, ^63^ One possible interpretation is that adequate MT can compensate for unfavorable BBD in terms of STM stability,^19^, ^58^, ^64^ even if it may not fully protect against future bone loss. Another explanation is that BBD may be more relevant to progressive hard tissue remodeling than to the position of the STM per se.^65^, ^66^ The weak and non‐significant correlation between BBD and MT indicates that these two variables are largely independent. Buccal bone defects can occur at sites with both thin and thick soft tissues, and vice versa, suggesting that bone position and MT represent distinct anatomical features with different etiologies and different impacts on clinical outcomes.

The subgroup analyses provide further nuance and have direct implications for both primary prevention and the management of existing esthetic complications. In implants without PSTD at *T*_0_, MT3 was the only significant predictor of future STM stability. This highlights the importance of assessing and, when needed, augmenting MT at or shortly after implant placement as a primary preventive strategy in the esthetic zone.^9^, ^27^, ^29^, ^67^ In contrast, in implants that already presented with PSTD at *T*_0_, the prognostic scenario was more complex. Although MT3 remained the most powerful predictor, other factors such as MT1, BBT, baseline PSTD depth, and metal exposure showed significant associations with the stability of the STM. This indicates that once a PSTD has developed, the biologic equilibrium appears to be disrupted, and the prognosis becomes dependent on a constellation of anatomical and clinical variables rather than on a single dominant factor. This multifactorial behavior mirrors what is observed in gingival recessions around natural teeth,^68^ where once the soft tissue defect has been established, the biological balance is disrupted and progression is more likely than spontaneous stabilization.^69^, ^70^ In the present cohort, the overall prevalence of PSTD increased from 51.1% at *T*_0_ to 74.4% at *T*_5_, with a 47.7% of the initially unaffected implants developing new defects during the observation period. Importantly, none of the implants with PSTD at baseline showed complete resolution over 5 years. These findings underscore the progressive and largely irreversible character of PSTDs once established.

From a clinical perspective, the data support two main principles. First, primary prevention through the establishment of adequate MT3 should be prioritized, particularly in high esthetic risk sites. This can be achieved by careful three‐dimensional implant positioning, appropriate prosthetic design, and early soft tissue augmentation when the phenotype is thin.^27^, ^56^, ^67^ Second, once PSTD is present, conservative monitoring is unlikely to lead to spontaneous improvement. Surgical intervention combining flap management with soft tissue grafting to increase MT appears to be the most rational approach to treat the defect, establish a protective soft tissue volume, and prevent further progression.^19^, ^58^, ^64^, ^71^

Several limitations should be considered when interpreting these findings. This was a single center study in a specific patient population with healthy implants in the maxillary esthetic zone, which may limit generalizability to other clinical scenarios, such as posterior sites, different implant systems, or patients with active peri‐implant disease. The observational design does not allow definitive causal inferences, and unmeasured confounders such as precise bucco‐lingual implant positioning, implant angulation, diameter, crown emergence profile, patient level behavioral factors, and history of bone augmentation at the implant site may have influenced the outcomes.^7^, ^8^, ^10^, ^59^ In addition, while the sample size was sufficient to detect strong associations and to perform ROC analysis, the quasi‐complete separation produced by MT3 prevented a stable multivariate model from being fitted, which restricts formal assessment of interactions between predictors. Despite these limitations, this study provides robust longitudinal evidence that MT3 is the critical soft tissue parameter governing the 5‐year stability of the peri‐implant STM at anterior implants. By identifying precise statistical and clinical thresholds, these findings expand the current understanding of peri‐implant soft tissue biology and support a shift toward a soft‐tissue‐centered framework for preventing and managing esthetic complications around implants.^27^, ^60^, ^72^ MT emerges as the primary therapeutic target and a decisive determinant of long‐term esthetic success, overshadowing the influence of other phenotype components once adequate MT is achieved. Future multicenter prospective studies should validate the proposed thresholds across broader populations, integrate hard tissue measurements and three‐dimensional implant positioning, and evaluate whether targeted soft tissue augmentation to reach the clinical stability threshold results in predictable long‐term stability of the STM.

## 5 CONCLUSIONS

Within the limitations of this 5‐year longitudinal assessment, baseline MT3 was identified as the dominant predictor of peri‐implant STM stability. A clinical stability threshold of ≥ 2.23 mm ensures 100% stability, and may be considered the critical MT for long‐term STM stability. MT3 overwhelmed all other predictors in a hierarchical model, with no synergistic effects observed. For implants without PSTD, MT3 is the sole determinant of STM stability, whereas the prognosis for implants with established PSTD is influenced by multiple interacting factors beyond MT3 alone.

From a clinical standpoint, these findings support the routine assessment of MT as part of the pre‐prosthetic or treatment planning evaluation of anterior implants. When MT3 falls below the identified safety threshold, soft tissue augmentation should be considered to reduce the risk of apical shift of the STM over the years. Implants presenting with PSTD at baseline and thin soft tissue phenotype should be considered for soft tissue reconstruction, as the deformity persists and may progress over time. Future prospective studies are needed to validate these thresholds and clinical recommendations.

## AUTHOR CONTRIBUTIONS

Lorenzo Tavelli, Shayan Barootchi, Hom‐Lay Wang, Istvan A. Urban, Martina Stefanini, and Giovanni Zucchelli participated in the study design. Lorenzo Tavelli, Hamoun Sabri, and Shayan Barootchi conducted clinical measures. Erfan Barootchi and Shayan Barootchi conducted statistical analyses. Lorenzo Tavelli and Shayan Barootchi led the writing of the manuscript. All authors reviewed the manuscript and approved its final version.

## CONFLICT OF INTEREST STATEMENT

The authors declare no conflicts of interest.

## Supporting information




**Supporting Information**: jper70159‐sup‐0001‐tableS1.docx

**Supporting Information**: jper70159‐sup‐0002‐tableS2.docx

**Supporting Information**: jper70159‐sup‐0003‐tableS3.docx

**Supporting Information**: jper70159‐sup‐0004‐tableS4.docx

**Supporting Information**: jper70159‐sup‐0005‐tableS5.docx

**Supporting Information**: jper70159‐sup‐0006‐figureS1.tiff

**Supporting Information**: jper70159‐sup‐0007‐SuppMat.docx

## Data Availability

Due to participant confidentiality, the full dataset is not publicly accessible; de‐identified data may be provided by the corresponding author upon reasonable request.

## References

[1] Chackartchi T , Romanos GE , Sculean A . Soft tissue‐related complications and management around dental implants. Periodontol 2000. 2019;81:124‐138. doi:10.1111/prd.12287 10.1111/prd.12287 31407443

[2] Wang II , Barootchi S , Tavelli L , Wang HL . The peri‐implant phenotype and implant esthetic complications. Contemporary overview. J Esthet Restor Dent. 2021;33:212‐223. doi:10.1111/jerd.12709 10.1111/jerd.12709 33459483

[3] Zucchelli G , Tavelli L , McGuire MK , et al. Autogenous soft tissue grafting for periodontal and peri‐implant plastic surgical reconstruction. J Periodontol. 2020;91:9‐16. doi:10.1002/JPER.19‐0350 10.1002/JPER.19-0350 31461778

[4] Anderson LE , Inglehart MR , El‐Kholy K , Eber R , Wang HL . Implant associated soft tissue defects in the anterior maxilla: a randomized control trial comparing subepithelial connective tissue graft and acellular dermal matrix allograft. Implant Dent. 2014;23:416‐425. 10.1097/ID.0000000000000122 25033346

[5] Zucchelli G , Tavelli L , Stefanini M , et al. Classification of facial peri‐implant soft tissue dehiscence/deficiencies at single implant sites in the esthetic zone. J Periodontol. 2019;90:1116‐1124. doi:10.1002/JPER.18‐0616 10.1002/JPER.18-0616 31087334

[6] Roccuzzo M , Gaudioso L , Bunino M , Dalmasso P . Surgical treatment of buccal soft tissue recessions around single implants: 1‐year results from a prospective pilot study. Clin Oral Implants Res. 2014;25:641‐646. doi:10.1111/clr.12149 10.1111/clr.12149 23582029

[7] Tavelli L , Barootchi S . Prevalence, incidence, risk, and protective factors for soft tissue dehiscences at implant sites in the absence of disease: an AO/AAP systematic review and meta‐regression analysis. J Periodontol. 2025;96:562‐586. doi:10.1002/JPER.24‐0119 10.1002/JPER.24-0119 PMC12273775 40489305

[8] Romandini M , Pedrinaci I , Lima C , Soldini MC , Araoz A , Sanz M . Prevalence and risk/protective indicators of buccal soft tissue dehiscence around dental implants. J Clin Periodontol. 2021;48:455‐463. doi:10.1111/jcpe.13417 10.1111/jcpe.13417 33378079

[9] Tavelli L , Heck T , De Souza AB , Stefanini M , Zucchelli G , Barootchi S . Implant esthetic complications: anatomical, prosthetic, and patient‐centered considerations for treatment. Int J Periodontics Restorative Dent. 2023;43:281‐288. 10.11607/prd.6538 37141083

[10] Sanz‐Martin I , Regidor E , Navarro J , Sanz‐Sanchez I , Sanz M , Ortiz‐Vigon A . Factors associated with the presence of peri‐implant buccal soft tissue dehiscences: a case‐control study. J Periodontol. 2020;91:1003‐1010. doi:10.1002/JPER.19‐0490 10.1002/JPER.19-0490 31977079

[11] Burkhardt R , Joss A , Lang NP . Soft tissue dehiscence coverage around endosseous implants: a prospective cohort study. Clin Oral Implants Res. 2008;19:451‐457. doi:10.1111/j.1600‐0501.2007.01497.x 10.1111/j.1600-0501.2007.01497.x 18371102

[12] Stefanini M , Marzadori M , Tavelli L , Bellone P , Zucchelli G . Peri‐implant papillae reconstruction at an esthetically failing implant. Int J Periodontics Restorative Dent. 2020;40:213‐222. doi:10.11607/prd.4296 10.11607/prd.4296 32032405

[13] Tavelli L , Majzoub J , Kauffmann F , et al. Coronally advanced flap versus tunnel technique for the treatment of peri‐implant soft tissue dehiscences with the connective tissue graft: a randomized, controlled clinical trial. J Clin Periodontol. 2023;50:980‐995. doi:10.1111/jcpe.13806 10.1111/jcpe.13806 36935199

[14] Mazzotti C , Stefanini M , Felice P , Bentivogli V , Mounssif I , Zucchelli G . Soft‐tissue dehiscence coverage at peri‐implant sites. Periodontol 2000. 2018;77:256‐272. doi:10.1111/prd.12220 10.1111/prd.12220 29473219

[15] Tavelli L , Barootchi S , Majzoub J , et al. Prevalence and risk indicators of midfacial peri‐implant soft tissue dehiscence at single site in the esthetic zone: a cross‐sectional clinical and ultrasonographic study. J Periodontol. 2022;93:857‐866. doi:10.1002/JPER.21‐0402 10.1002/JPER.21-0402 34787892

[16] von Elm E , Altman DG , Egger M , et al. The strengthening the reporting of observational studies in epidemiology (STROBE) statement: guidelines for reporting observational studies. J Clin Epidemiol. 2008;61:344‐349. doi:10.1016/j.jclinepi.2007.11.008 10.1016/j.jclinepi.2007.11.008 18313558

[17] Berglundh T , Armitage G , Araujo MG , et al. Peri‐implant diseases and conditions: consensus report of workgroup 4 of the 2017 World Workshop on the classification of periodontal and peri‐implant diseases and conditions. J Clin Periodontol. 2018;45(Suppl 20):S286Z‐S291. 10.1111/jcpe.12957 29926491

[18] Tavelli L , Barootchi S , Majzoub J , Siqueira R , Mendonca G , Wang HL . Volumetric changes at implant sites: a systematic appraisal of traditional methods and optical scanning‐based digital technologies. J Clin Periodontol. 2021;48:315‐334. doi:10.1111/jcpe.13401 10.1111/jcpe.13401 33151586

[19] Tavelli L , Majzoub J , Kauffmann F , et al. Coronally Advanced flap vs Tunnel technique for the treatment of peri‐implant soft tissue dehiscences with the connective tissue graft: a randomized, controlled, clinical trial. J Clin Periodontol. 2023;50(7):980‐995. doi:10.1111/jcpe.13806 10.1111/jcpe.13806 36935199

[20] Barootchi S , Chan HL , Namazi SS , Wang HL , Kripfgans OD . Ultrasonographic characterization of lingual structures pertinent to oral, periodontal, and implant surgery. Clin Oral Implants Res. 2020;31(4):352‐359. doi:10.1111/clr.13573 10.1111/clr.13573 PMC7959268 31925829

[21] Chan HL , Wang HL , Fowlkes JB , Giannobile WV , Kripfgans OD . Non‐ionizing real‐time ultrasonography in implant and oral surgery: a feasibility study. Clin Oral Implants Res. 2017;28:341‐347. doi:10.1111/clr.12805 10.1111/clr.12805 PMC11992668 26992276

[22] Chan HL , Kripfgans OD . Ultrasonography for diagnosis of peri‐implant diseases and conditions: a detailed scanning protocol and case demonstration. Dentomaxillofac Radiol. 2020:20190445. doi:10.1259/dmfr.20190445 10.1259/dmfr.20190445 PMC7549532 32017634

[23] Tavelli L , Barootchi S , Majzoub J , et al. Ultrasonographic tissue perfusion analysis at implant and palatal donor sites following soft tissue augmentation: a clinical pilot study. J Clin Periodontol. 2021;48(4):602‐614. doi:10.1111/jcpe.13424 10.1111/jcpe.13424 PMC8058327 33465812

[24] Barootchi S , Tavelli L , Majzoub J , Chan HL , Wang HL , Kripfgans OD . Ultrasonographic tissue perfusion in peri‐implant health and disease. J Dent Res. 2021;101(3):278‐285. 10.1177/00220345211035684 PMC8982012 34515570

[25] Monje A , Chappuis V , Monje F , et al. The critical peri‐implant buccal bone wall thickness revisited: an experimental study in the beagle dog. Int J Oral Maxillofac Implants. 2019;34:1328‐1336. doi:10.11607/jomi.7657 10.11607/jomi.7657 31532826

[26] Spray JR , Black CG , Morris HF , Ochi S . The influence of bone thickness on facial marginal bone response: stage 1 placement through stage 2 uncovering. Ann Periodontol. 2000;5:119‐128. doi:10.1902/annals.2000.5.1.119 10.1902/annals.2000.5.1.119 11885170

[27] Tavelli L , Barootchi S , Akhondi S , et al. Long‐term stability of soft tissue augmentative procedures at implant sites. Periodontol 2000 Published online October 28, 2025. doi:10.1111/prd.70016 10.1111/prd.70016 41147360

[28] Tavelli L , Barootchi S , Avila‐Ortiz G , Urban IA , Giannobile WV , Wang HL . Peri‐implant soft tissue phenotype modification and its impact on peri‐implant health: a systematic review and network meta‐analysis. J Periodontol. 2021;92:21‐44. doi:10.1002/JPER.19‐0716 10.1002/JPER.19-0716 32710810

[29] Tavelli L , Thoma D , Galarraga‐Vinueza ME , Romandini M , Barootchi S . Soft tissue substitutes: current biomaterials and indications at teeth and implant sites. J Periodontal Res. 2025. doi:10.1111/jre.70066 10.1111/jre.70066 41474175

[30] Sabri H , Hazrati P , Tavelli L , et al. Impact of peri‐implant phenotype on implant therapy outcomes: a 5‐Year cohort analysis on soft tissue‐level implants. J Clin Periodontol. 2026;53(5):394‐406. doi:10.1111/jcpe.70062 10.1111/jcpe.70062 PMC12890457 41387987

[31] Chappuis V , Engel O , Reyes M , Shahim K , Nolte LP , Buser D . Ridge alterations post‐extraction in the esthetic zone: a 3D analysis with CBCT. J Dent Res. 2013;92:195S‐201S. doi:10.1177/0022034513506713 10.1177/0022034513506713 PMC3860068 24158340

[32] Merheb J , Quirynen M , Teughels W . Critical buccal bone dimensions along implants. Periodontol 2000. 2014;66:97‐105. doi:10.1111/prd.12042 10.1111/prd.12042 25123763

[33] Cicciù M , Pratella U , Fiorillo L , et al. Influence of buccal and palatal bone thickness on post‐surgical marginal bone changes around implants placed in posterior maxilla: a multi‐centre prospective study. BMC Oral Health. 2023;23:309. 10.1186/s12903-023-02991-3 PMC10204312 37217911

[34] Wennstrom JL , Derks J . Is there a need for keratinized mucosa around implants to maintain health and tissue stability?. Clin Oral Implants Res. 2012;23(Suppl 6):136‐146. doi:10.1111/j.1600‐0501.2012.02540.x 10.1111/j.1600-0501.2012.02540.x 23062138

[35] Warrer K , Buser D , Lang NP , Karring T . Plaque‐induced peri‐implantitis in the presence or absence of keratinized mucosa. An experimental study in monkeys. Clin Oral Implants Res. 1995;6:131‐138. doi:10.1034/j.1600‐0501.1995.060301.x 10.1034/j.1600-0501.1995.060301.x 7578788

[36] Mancini L , Strauss FJ , Lim HC , et al. Impact of keratinized mucosa on implant‐health related parameters: a 10‐year prospective re‐analysis study. Clin Implant Dent Relat Res. 2024;26:554‐563. doi:10.1111/cid.13314 10.1111/cid.13314 38419210

[37] Roccuzzo M , Grasso G , Dalmasso P . Keratinized mucosa around implants in partially edentulous posterior mandible: 10‐year results of a prospective comparative study. Clin Oral Implants Res. 2016;27:491‐496. doi:10.1111/clr.12563 10.1111/clr.12563 25706508

[38] Monje A , Blasi G . Significance of keratinized mucosa/gingiva on peri‐implant and adjacent periodontal conditions in erratic maintenance compliers. J Periodontol. 2019;90:445‐453. doi:10.1002/JPER.18‐0471 10.1002/JPER.18-0471 30461016

[39] Isler SC , Romandini M , Akca G , et al. Soft‐tissue phenotype as a risk indicator of peri‐implantitis and peri‐implant soft‐tissue dehiscence‐A cross‐sectional study. J Clin Periodontol. 2024;51:1443‐1457. doi:10.1111/jcpe.14059 10.1111/jcpe.14059 39189550

[40] Perussolo J , Souza AB , Matarazzo F , Oliveira RP , Araujo MG . Influence of the keratinized mucosa on the stability of peri‐implant tissues and brushing discomfort: a 4‐year follow‐up study. Clin Oral Implants Res. 2018;29:1177‐1185. doi:10.1111/clr.13381 10.1111/clr.13381 30346630

[41] Lin GH , Chan HL , Wang HL . The significance of keratinized mucosa on implant health: a systematic review. J Periodontol. 2013;84:1755‐1767. doi:10.1902/jop.2013.120688 10.1902/jop.2013.120688 23451989

[42] Galarraga‐Vinueza ME , Tavelli L . Soft tissue features of peri‐implant diseases and related treatment. Clin Implant Dent Relat Res. 2023;25:661‐681. doi:10.1111/cid.13156 10.1111/cid.13156 36444772

[43] Liu Z , Li C , Liu Y , et al. The clinical significance and application of the peri‐implant phenotype in dental implant surgery: a narrative review. Ann Transl Med. 2023;11:351. doi:10.21037/atm‐23‐1752 10.21037/atm-23-1752 PMC10477659 37675312

[44] Linkevicius T , Apse P , Grybauskas S , Puisys A . The influence of soft tissue thickness on crestal bone changes around implants: a 1‐year prospective controlled clinical trial. Int J Oral Maxillofac Implants. 2009;24:712‐719. 19885413

[45] Linkevicius T , Puisys A , Steigmann M , Vindasiute E , Linkeviciene L . Influence of vertical soft tissue thickness on crestal bone changes around implants with platform switching: a comparative clinical study. Clin Implant Dent Relat Res. 2015;17:1228‐1236. doi:10.1111/cid.12222 10.1111/cid.12222 24673875

[46] Puisys A , Linkevicius T . The influence of mucosal tissue thickening on crestal bone stability around bone‐level implants. A prospective controlled clinical trial. Clin Oral Implants Res. 2015;26:123‐129. doi:10.1111/clr.12301 10.1111/clr.12301 24313250

[47] Puisys A , Vindasiute‐Narbute E , Razukevicius D , Akhondi S , Gallucci GO , Pedrinaci I . Influence of mucosal tissue height on implant crestal bone: a 10‐year follow‐up of a controlled clinical trial. J Dent. 2024;148:105224. doi:10.1016/j.jdent.2024.105224 10.1016/j.jdent.2024.105224 38955258

[48] Cosgarea R , Gasparik C , Dudea D , Culic B , Dannewitz B , Sculean A . Peri‐implant soft tissue colour around titanium and zirconia abutments: a prospective randomized controlled clinical study. Clin Oral Implants Res. 2015;26:537‐544. doi:10.1111/clr.12440 10.1111/clr.12440 24961535

[49] Jun SH , Ahn JS , Chang BM , Lee JD , Ryu JJ , Kwon JJ . In vivo measurements of human gingival translucency parameters. Int J Periodontics Restorative Dent. 2013;33:427‐434. doi:10.11607/prd.1497 10.11607/prd.1497 23820703

[50] Lops D , Stellini E , Sbricoli L , Cea N , Romeo E , Bressan E . Influence of abutment material on peri‐implant soft tissues in anterior areas with thin gingival biotype: a multicentric prospective study. Clin Oral Implants Res. 2017;28:1263‐1268. doi:10.1111/clr.12952 10.1111/clr.12952 27699895

[51] Jung RE , Sailer I , Hammerle CH , Attin T , Schmidlin P . In vitro color changes of soft tissues caused by restorative materials. Int J Periodontics Restorative Dent. 2007;27:251‐257. 17694948

[52] Mancini L , Barootchi S , Thoma DS , et al. The peri‐implant mucosa color: a systematic appraisal of methods for its assessment and clinical significance. Clin Implant Dent Relat Res. 2023;25(2):224‐240. doi:10.1111/cid.13180 10.1111/cid.13180 36646440

[53] Garaicoa‐Pazmino C , Mendonca G , Ou A , et al. Impact of mucosal phenotype on marginal bone levels around tissue level implants: a prospective controlled trial. J Periodontol. 2021;92:771‐783. doi:10.1002/JPER.20‐0458 10.1002/JPER.20-0458 33107977

[54] Bhat PR , Thakur SL , Kulkarni SS . The influence of soft tissue biotype on the marginal bone changes around dental implants: a 1‐year prospective clinico‐radiological study. J Indian Soc Periodontol. 2015;19:640‐644. 10.4103/0972-124X.168489 PMC4753708 26941514

[55] Thoma DS , Naenni N , Figuero E , et al. Effects of soft tissue augmentation procedures on peri‐implant health or disease: a systematic review and meta‐analysis. Clin Oral Implants Res. 2018;29(Suppl 15):32‐49. doi:10.1111/clr.13114 10.1111/clr.13114 29498129

[56] Puzio M , Hadzik J , Blaszczyszyn A , Gedrange T , Dominiak M . Soft tissue augmentation around dental implants with connective tissue graft (CTG) and xenogenic collagen matrix (XCM). 1‐year randomized control trail. Ann Anat. 2020;230:151484. doi:10.1016/j.aanat.2020.151484 10.1016/j.aanat.2020.151484 32205206

[57] Zucchelli G , Sharma P , Mounssif I . Esthetics in periodontics and implantology. Periodontol 2000. 2018;77:7‐18. doi:10.1111/prd.12207 10.1111/prd.12207 29508445

[58] Roccuzzo A , Mancini L , Marruganti C , et al. Long‐term treatment outcomes of single maxillary buccal peri‐implant soft tissue dehiscences: a 10‐year prospective study. Clin Implant Dent Relat Res. 2024;26:150‐157. doi:10.1111/cid.13273 10.1111/cid.13273 37691144

[59] Siegenthaler M , Strauss FJ , Gamper F , Hammerle CHF , Jung RE , Thoma DS . Anterior implant restorations with a convex emergence profile increase the frequency of recession: 12‐month results of a randomized controlled clinical trial. J Clin Periodontol. 2022;49:1145‐1157. doi:10.1111/jcpe.13696 10.1111/jcpe.13696 PMC9804465 35817419

[60] Jensen SS , Aghaloo T , Jung RE , et al. Group 1 ITI Consensus Report: the role of bone dimensions and soft tissue augmentation procedures on the stability of clinical, radiographic, and patient‐reported outcomes of implant treatment. Clin Oral Implants Res. 2023(Suppl 34 26):43‐49. doi:10.1111/clr.14154 10.1111/clr.14154 37750519

[61] Jung RE , Becker K , Bienz SP , et al. Effect of peri‐implant mucosal thickness on esthetic outcomes and the efficacy of soft tissue augmentation procedures: consensus report of group 2 of the SEPA/DGI/OF workshop. Clin Oral Implants Res. 2022(Suppl 33 23):100‐108. doi:10.1111/clr.13955 10.1111/clr.13955 PMC9543632 35763020

[62] Evans CDJ , ST Chen . Esthetic outcomes of immediate implant placements. Clin Oral Implants Res. 2008;19:73‐80. doi:10.1111/j.1600‐0501.2007.01413.x 10.1111/j.1600-0501.2007.01413.x 17956569

[63] Kan JY , Rungcharassaeng K , Sclar A , Lozada JL . Effects of the facial osseous defect morphology on gingival dynamics after immediate tooth replacement and guided bone regeneration: 1‐year results. J Oral Maxillofac Surg. 2007;65:13‐19. doi:10.1016/j.joms.2007.04.006 10.1016/j.joms.2007.04.006 17586345

[64] Zucchelli G , Felice P , Mazzotti C , et al. 5‐year outcomes after coverage of soft tissue dehiscence around single implants: a prospective cohort study. Eur J Oral Implantol. 2018;11:215‐224. 29806668

[65] Sanz‐Martin I , Regidor E , Cosyn J , Wiedemeier DB , Thoma DS . Buccal soft tissue dehiscence defects at dental implants‐associated factors and frequency of occurrence: a systematic review and meta‐analysis. Clin Oral Implants Res. 2022(Suppl 33 23):109‐124. doi:10.1111/clr.13888 10.1111/clr.13888 35763025

[66] Monje A , Roccuzzo A , Buser D , Wang HL . Influence of buccal bone wall thickness on the peri‐implant hard and soft tissue dimensional changes: a systematic review. Clin Oral Implants Res. 2023;34:157‐176. doi:10.1111/clr.14029 10.1111/clr.14029 36626118

[67] Mancini L , Simeone D , Roccuzzo A , Strauss FJ , Marchetti E . Timing of soft tissue augmentation around implants: a clinical review and decision tree. Int J Oral Implantol (Berl). 2023;16:289‐302. 37994817

[68] Mascardo KC , Tomack J , Chen CY , et al. Risk indicators for gingival recession in the esthetic zone: a cross‐sectional clinical, tomographic, and ultrasonographic study. J Periodontol. 2024;95:432‐443. doi:10.1002/JPER.23‐0357 10.1002/JPER.23-0357 38196327

[69] Rios FS , Costa RSA , Wagner TP , et al. Incidence and progression of gingival recession over 4 years: a population‐based longitudinal study. J Clin Periodontol. 2021;48:114‐125. doi:10.1111/jcpe.13383 10.1111/jcpe.13383 33015887

[70] Chambrone L , Tatakis DN . Long‐term outcomes of untreated buccal gingival recessions: a systematic review and meta‐analysis. J Periodontol. 2016;87:796‐808. doi:10.1902/jop.2016.150625 10.1902/jop.2016.150625 26878749

[71] Tavelli L , Akhondi S , Vinueza MEG , Mancini L , Lanis A , Barootchi S . The tunneled coronally advanced flap for vertical soft tissue reconstruction and papilla augmentation at implant sites (verTCAF). Int J Periodontics Restorative Dent. 2025;0:1‐26. doi:10.11607/prd.7619 10.11607/prd.7619 40198777

[72] Thoma DS , Cosyn J , Fickl S , et al. Soft tissue management at implants: summary and consensus statements of group 2. The 6th EAO Consensus Conference 2021. Clin Oral Implants Res. 2021;32(Suppl 21):174‐180. doi:10.1111/clr.13798 10.1111/clr.13798 PMC8596754 34145925

