# Supplementary material for: Protective factors and critical mucosal thickness for the stability of the peri‐implant soft tissue margin over 5 years: A longitudinal cohort study
Source: J Periodontol. 2026 Jul 12;97(10):2136–49. doi: 10.1002/jper.70159 (PMC13743502; doi:10.1002/jper.70159)
Supplement: Supplementary file 1 [file JPER-97-2136-s001.docx]

Supplementary Table 2. Univariate linear regression analysis evaluating baseline predictors of PSTD depth change at 5 years. The coefficient represents the changes in PSTD depth for each 1-unit increase in the predictor. MT3 alone explains 65.2% of the variance in PSTD change over 5 years. For every 1 mm increase in baseline MT3, the apical shift of the STM is reduced by 0.51 mm.

| Predictor (at baseline) | Coefficient (β) | 95% CI | R² | p-value |
| --- | --- | --- | --- | --- |
| MT3 (mm) | -0.508 | [-0.572, -0.444] | 0.652 | <0.0001* |
| MT1 (mm) | -0.224 | [-0.364, -0.084] | 0.071 | 0.002* |
| KM width (mm) | -0.028 | [-0.066, 0.010] | 0.016 | 0.149 |
| BBT (mm) | -0.024 | [-0.147, 0.099] | 0.001 | 0.700 |
| BBD (mm) | 0.017 | [-0.023, 0.057] | 0.006 | 0.391 |
| PSTD Depth (mm) | 0.013 | [-0.015, 0.041] | 0.006 | 0.362 |
| Years in Function | -0.0004 | [-0.019, 0.018] | 0.00001 | 0.968 |

**Table legend.** BBD: buccal bone dehiscence. BBT: buccal bone thickness. CI: confidence interval. KM: keratinized mucosa. MT1: mucosal thickness assessed 1 mm apical to the soft tissue margin. MT3: mucosal thickness assessed 3 mm apical to the soft tissue margin. PSTD: peri-implant soft tissue dehiscence. STM: soft tissue margin. *denotes statistically significance.
