# Supplementary material for: Protective factors and critical mucosal thickness for the stability of the peri‐implant soft tissue margin over 5 years: A longitudinal cohort study
Source: J Periodontol. 2026 Jul 12;97(10):2136–49. doi: 10.1002/jper.70159 (PMC13743502; doi:10.1002/jper.70159)
Supplement: Supplementary file 2 [file JPER-97-2136-s002.docx]

**Supplementary Table 5.** Baseline predictors of 5-year STM stability stratified by initial PSTD status (non-PSTD vs PSTD groups). A) non-PSTD group. B) PSTD group. Overall, MT3 is the only significant predictor of future stability at implant sites initially diagnosed without PSTD. For implants diagnosed with PSTD already, while MT3 remains the most powerful predictor for STM stability, multiple other factors were also significant, such as MT1, BBT, PSTD and metal exposure.

A) Non-PSTD group

| Variable (at baseline) | Stable Group (n=34)  Mean ± SD | Not Stable Group (n=31)  Mean ± SD | p-value |
| --- | --- | --- | --- |
| MT3 (mm) | 2.76 ± 0.44 | 1.39 ± 0.47 | <0.0001* |
| MT1 (mm) | 1.57 ± 0.56 | 1.31 ± 0.57 | 0.060 |
| KM width (mm) | 4.35 ± 1.78 | 4.60 ± 1.64 | 0.441 |
| BBT (mm) | 1.48 ± 0.68 | 1.51 ± 0.74 | 0.967 |
| BBD (mm) | 2.10 ± 1.56 | 1.58 ± 0.76 | 0.148 |
| PSTD Depth (mm) | 0.00 ± 0.00 | 0.00 ± 0.00 | 1.000 |
| Years in Function | 5.32 ± 1.64 | 4.97 ± 1.06 | 0.312 |
| Presence of adjacent implant (no) | 27 (79.4%) | 34 (100%) | 0.056 |
| Presence of adjacent implant (yes) | 4 (11.8%) | 0 (0%) |  |

**Table legend.** BBD: buccal bone dehiscence. BBT: buccal bone thickness. KM: keratinized mucosa. MT1: mucosal thickness assessed 1 mm apical to the soft tissue margin. MT3: mucosal thickness assessed 3 mm apical to the soft tissue margin. PSTD: peri-implant soft tissue dehiscence. SD: standard deviation. STM: soft tissue margin. *denotes statistically significance. Mann-Whitney U test was used for continuous variables, while Fisher’s exact test for categorical parameters.

B) PSTD group

| Variable (at baseline) | Stable Group (n=14)  Mean ± SD | Not Stable Group (n=54)  Mean ± SD | p-value |
| --- | --- | --- | --- |
| MT3 (mm) | 2.39 ± 0.32 | 1.40 ± 0.36 | <0.0001* |
| MT1 (mm) | 1.20 ± 0.35 | 0.89 ± 0.37 | 0.005* |
| KM width (mm) | 3.29 ± 2.48 | 1.94 ± 1.33 | 0.087 |
| BBT (mm) | 1.22 ± 0.57 | 0.85 ± 0.33 | 0.029* |
| BBD (mm) | 3.26 ± 2.29 | 3.62 ± 2.16 | 0.688 |
| PSTD Depth (mm) | 1.36 ± 0.53 | 2.48 ± 4.05 | 0.016* |
| Years in Function | 10.07 ± 5.84 | 9.59 ± 4.47 | 0.988 |
| Presence of adjacent implant (no) | 8 (57.1%) | 29 (53.7%) | 1.000 |
| Presence of adjacent implant (yes) | 6 (42.9%) | 25 (46.3%) |  |
| Metal exposure (no) | 13 (92.9%) | 25 (46.3%) | 0.0002* |
| Metal exposure (yes) | 1 (7.1%) | 29 (53.7%) |  |

**Table legend.** BBD: buccal bone dehiscence. BBT: buccal bone thickness. KM: keratinized mucosa. MT1: mucosal thickness assessed 1 mm apical to the soft tissue margin. MT3: mucosal thickness assessed 3 mm apical to the soft tissue margin. PSTD: peri-implant soft tissue dehiscence. SD: standard deviation. STM: soft tissue margin. *denotes statistically significance. Mann-Whitney U test was used for continuous variables, while Fisher’s exact test for categorical parameters.
