# Supplementary material for: Protective factors and critical mucosal thickness for the stability of the peri‐implant soft tissue margin over 5 years: A longitudinal cohort study
Source: J Periodontol. 2026 Jul 12;97(10):2136–49. doi: 10.1002/jper.70159 (PMC13743502; doi:10.1002/jper.70159)
Supplement: Supplementary file 3 [file JPER-97-2136-s003.docx]

**Protective Factors and Critical Mucosal Thickness for the Stability of the Peri-implant Soft Tissue Margin over 5 years: A Longitudinal Cohort Study**

Lorenzo Tavelli, Hamoun Sabri, Erfan Barootchi, Martina Stefanini, Giovanni Zucchelli, Hom-Lay Wang, Istvan A. Urban, and Shayan Barootchi

**Supplementary Appendix**

**Setting and participants**

Subjects with one or more healthy dental implants in the esthetic area (from the right first premolar to the left first premolar) were identified and recruited at the Graduate Periodontics clinic, University of Michigan School of Dentistry, Ann Arbor, if they met the following inclusion criteria: 1) systemically and periodontally healthy subjects, 2) having at least one anterior dental implant with two adjacent natural teeth and/or dental implants, 3) dental implant(s) diagnosed as healthy (“absence of erythema, bleeding on probing, swelling and suppuration”^1, 2^), 4) dental implants rehabilitated with a single implant-supported crown, 5) loading time of at least 24 months ^3^ , 6) presence of the homologous contralateral natural tooth, 7) available information regarding implant characteristics and 8) patients willing to provide an informed consent and attend the study. On the other hand, exclusion criteria were: 1) Multiple adjacent dental implants with PSTDs, 2) implants in the second premolar or molar region, 3) one or two adjacent edentulous area, 4) implant(s) restored with three (or more)-unit fixed bridges, single crown with cantilever or removable prosthesis, 5) diagnosis of periodontitis, 6) any confirmed peri-implant disease ^1, 2^, 6) documented history of peri-implantitis or previous surgical procedures at the implant site, 7) previous soft tissue graft at the implant site and 8) missing information on the implant characteristics. The same cohort was recalled 5 years after the initial visit. The clinical examinations were conducted two calibrated clinicians (S.B. and L.T.), with intra- and inter-examiner reliability confirmed prior to data collection.

The implants assessed in this study were placed either at the same institution or external facilities. Consequently, implant surgeries were conducted by different providers, while the periodontal and peri-implant maintenance procedures were all performed by the dental hygienists of the Graduate Periodontics clinic at University of Michigan.

**Data collection and clinical measurements**

At each study visit (baseline [T_0_] and at the 5-year follow-up [T_5_]), patient demographics, implant characteristics, and adverse events at the study site during the last 5 years (e.g., changes in implant health status) were recorded. In addition, the following clinical measures were assessed:

- Presence or absence of PSTD, defined as the apical shift of the STM compared to the ideal position of the gingival margin of the homologous contralateral natural tooth ^4^. PSTD change between T_0_ and T_5_ was assessed as the apico-coronal shift of the peri-implant soft tissue margin through superimposing digital impressions ^14, 15^.
- PSTD classification, in terms of class (I, II or III/IV) and subclass (a, b or c).
- Presence or absence of an implant-supported crown longer than the clinical crown of the homologous contralateral natural tooth
- Presence or absence of the exposure of the abutment and/or implant fixture to the oral cavity
- Presence of adjacent (mesial/distal) implants
- Probing depth (PD) using a periodontal probe
- KM width at the midfacial aspect

**Ultrasonographic image acquisition and measurements**

The ultrasound equipment setup and the scanning procedures have been previously described in detail ^5-9^. Single image frames (“still images”) at the mid-facial aspect of the implant(s) of interest were obtained in “B-mode”, and then exported in the Digital Imaging and Communications in Medicine (DICOM) format. The ultrasound probe was oriented perpendicular to the occlusal plane and parallel to the long axis of the implant at its midfacial aspect ^7, 8^.

The following measurements were computed using a commercially available software package (Horos v4.0.0, www.horosproject.org), as previously described ^6-12^ and were carried out by a single experienced and precalibrated examiner:

- Mucosal thickness (MT): horizontal thickness of the peri-implant soft tissue, calculated as the distance between the STM and the abutment/implant fixture/buccal bone on a line parallel to the long axis of the implant body in the mid-facial scan. MT was measured at 1 and 3 mm (MT1 and MT3, respectively) from the STM.
- Peri-implant buccal bone distance (BBD): Distance between the implant platform and the peri-implant bone crest evaluated on a line parallel to the long axis of the implant body in the mid-facial scan.
- Peri-implant buccal bone thickness (BBT): evaluated 0.5 mm apical to the bone crest as the distance between the peri-implant crestal bone and a line parallel to the long axis of the implant body in the mid-facial scan.

**Statistical Analysis**

The implant was the unit of analysis. Descriptive statistics were calculated for all baseline and 5-year parameters, with continuous variables reported as mean ± standard deviation and categorical variables as frequencies and percentages.

**Predictor Analysis**

To identify baseline (T0) parameters associated with the 5-year stability of the peri-implant soft tissue margin, a univariate analysis was conducted. For continuous variables (e.g., MT1, MT3, keratinized tissue width), simple logistic regression was used to calculate the Odds Ratio (OR) and 95% confidence interval (CI) for each 1-unit increase in the predictor. For categorical variables (e.g., initial diagnosis, presence of metal exposure), Chi-square or Fisher's exact tests were employed. As this is a longitudinal cohort, any baseline variable significantly associated with the 5-year outcome was interpreted as a potential predictor of stability.

**Critical Threshold Identification**

For the most determinant baseline parameters identified in the univariate analysis, Receiver Operating Characteristic (ROC) curve analysis was performed to assess their predictive power and identify optimal clinical thresholds. The Area Under the Curve (AUC) was calculated to assess the overall discriminatory ability. The optimal statistical cutoff was identified using the Youden Index (J = Sensitivity + Specificity - 1). A clinical certainty threshold was also determined by identifying the minimum value above which 100% of implants maintained a stable position of the soft tissue margin.

**Multi-level Analysis**

A multi-level logistic regression model was planned to determine the independent contribution of each significant predictor. However, this model could not be computed because baseline MT3 produced quasi-complete separation of the outcome groups. This statistical phenomenon occurs when a predictor variable so perfectly distinguishes the outcome groups that it prevents the model from estimating additional independent effects.

**Linear Regression for Tissue Change**

To quantify the impact of baseline predictors on the magnitude of soft tissue change, linear regression analysis was performed using the 5-year change in PSTD depth as the outcome variable. Both univariate and multivariate models were constructed to assess the independent contribution of each predictor.

**Interaction Analysis**

To investigate potential synergistic or antagonistic effects, interaction terms were tested for key predictor combinations (MT3 × KT, MT3 × PBT, MT3 × BBD) using logistic regression models with likelihood ratio tests.

**Correlation Analysis**

Pearson correlation coefficients were calculated to assess the relationships among baseline anatomical variables (MT1, MT3, KT, PBT, BBD), with the aim of determining whether these predictors represent distinct or overlapping anatomical features.

**Subgroup Analysis**

To determine whether predictors of stability differed based on initial tissue condition, separate analyses were performed for implants that started with PSTD (n=68) versus those that started without PSTD (Non-PSTD, n=65). Mann-Whitney U tests were used for continuous variables and Fisher's exact test for categorical variables.

**Esthetic Outcomes Analysis**

Patient-reported esthetic outcomes were assessed using a Visual Analog Scale (EST VAS) at 5 years. Multivariate linear regression was used to identify predictors of esthetic perception, controlling for PSTD depth at 5 years. Among patients with PSTD, factors associated with willingness to pursue treatment were analyzed using Mann-Whitney U tests and Fisher's exact tests.

**Longitudinal Comparison**

To assess changes in clinical and anatomical parameters over time, paired comparisons between baseline and 5-year measurements were performed using Wilcoxon signed-rank tests. Between-group comparisons (Non-PSTD vs PSTD, classified by baseline diagnosis) were conducted using Mann-Whitney U tests at each time point.

All statistical analyses were performed using RStudio (version 3.6.0, RStudio, Boston, MA, USA). A p-value < 0.05 was considered statistically significant.

REFERENCES

1.	Renvert S, Persson GR, Pirih FQ, Camargo PM. Peri-implant health, peri-implant mucositis, and peri-implantitis: Case definitions and diagnostic considerations. *J Clin Periodontol* 2018;45 Suppl 20:S278-S285.

2.	Berglundh T, Armitage G, Araujo MG, et al. Peri-implant diseases and conditions: Consensus report of workgroup 4 of the 2017 World Workshop on the Classification of Periodontal and Peri-Implant Diseases and Conditions. *J Periodontol* 2018;89 Suppl 1:S313-S318.

3.	Sanz-Martin I, Regidor E, Navarro J, Sanz-Sanchez I, Sanz M, Ortiz-Vigon A. Factors associated with the presence of peri-implant buccal soft tissue dehiscences: A case-control study. *J Periodontol* 2020.

4.	Zucchelli G, Tavelli L, Stefanini M, et al. Classification of facial peri-implant soft tissue dehiscence/deficiencies at single implant sites in the esthetic zone. *J Periodontol* 2019;90:1116-1124.

5.	Barootchi S, Chan HL, Namazi SS, Wang HL, Kripfgans OD. Ultrasonographic characterization of lingual structures pertinent to oral, periodontal, and implant surgery. *Clin Oral Implants Res* 2020.

6.	Chan HL, Wang HL, Fowlkes JB, Giannobile WV, Kripfgans OD. Non-ionizing real-time ultrasonography in implant and oral surgery: A feasibility study. *Clin Oral Implants Res* 2017;28:341-347.

7.	Chan HL, Kripfgans OD. Ultrasonography for diagnosis of peri-implant diseases and conditions: a detailed scanning protocol and case demonstration. *Dentomaxillofac Radiol* 2020:20190445.

8.	Tavelli L, Barootchi S, Majzoub J, et al. Ultrasonographic Tissue Perfusion Analysis at Implant and Palatal Donor Sites Following Soft Tissue Augmentation: A Clinical Pilot Study. *J Clin Periodontol* 2021.

9.	Barootchi S, Tavelli L, Majzoub J, Chan HL, Wang HL, Kripfgans OD. Ultrasonographic Tissue Perfusion in Peri-implant Health and Disease. *J Dent Res* 2021:220345211035684.

10.	Tattan M, Sinjab K, Lee E, et al. Ultrasonography for chairside evaluation of periodontal structures: A pilot study. *J Periodontol* 2019.

11.	Chan HL, Sinjab K, Li J, Chen Z, Wang HL, Kripfgans OD. Ultrasonography for noninvasive and real-time evaluation of peri-implant tissue dimensions. *J Clin Periodontol* 2018;45:986-995.

12.	Barootchi S, Chan HL, Namazi SS, Wang HL, Kripfgans OD. Ultrasonographic characterization of lingual structures pertinent to oral, periodontal, and implant surgery. *Clin Oral Implants Res* 2020;31:352-359.
