# Supplementary material for: Protective factors and critical mucosal thickness for the stability of the peri‐implant soft tissue margin over 5 years: A longitudinal cohort study
Source: J Periodontol. 2026 Jul 12;97(10):2136–49. doi: 10.1002/jper.70159 (PMC13743502; doi:10.1002/jper.70159)
Supplement: Supplementary file 4 [file JPER-97-2136-s004.docx]

Supplementary Table 3. Multi-level linear regression model evaluating baseline MT1 and MT3 as predictors of PSTD depth change at 5 years. The coefficient represents the changes in PSTD depth for each 1-unit increase in the predictor, adjusting for other variables in the model. MT3 alone explains 65.2% of the variance in PSTD change over 5 years. The analysis demonstrates that measuring MT3 is sufficient for predicting long-term STM stability, while measuring MT1 provides no additional independent predictive value once MT3 is known.

| Predictor | Coefficient (β) | Standard Error | t-value | p-value | 95% CI |
| --- | --- | --- | --- | --- | --- |
| Intercept | 1.419 | 0.071 | 20.034 | <0.0001* | [1.279, 1.559] |
| MT1 (mm) | 0.041 | 0.047 | 0.879 | 0.381 | [-0.052, 0.134] |
| MT3 (mm) | -0.520 | 0.035 | -14.803 | <0.0001* | [-0.589, -0.450] |

**Table legend.** CI: confidence interval. MT1: mucosal thickness assessed 1 mm apical to the soft tissue margin. MT3: mucosal thickness assessed 3 mm apical to the soft tissue margin. PSTD: peri-implant soft tissue dehiscence. STM: soft tissue margin. *denotes statistically significance.
