# Supplementary material for: Protective factors and critical mucosal thickness for the stability of the peri‐implant soft tissue margin over 5 years: A longitudinal cohort study
Source: J Periodontol. 2026 Jul 12;97(10):2136–49. doi: 10.1002/jper.70159 (PMC13743502; doi:10.1002/jper.70159)
Supplement: Supplementary file 5 [file JPER-97-2136-s006.docx]

**Supplementary Table 1**. The three clinical zones of risk for apical shift of the STM based on baseline MT3

| MT3 Zone | Baseline MT3 Range | Total Implants (N) | Stable Implants (n) | 5-Year STM Stability Rate |
| --- | --- | --- | --- | --- |
| High Risk Zone | < 2.01 mm | 85 | 0 | 0.0% |
| Gray Zone | 2.01 - 2.23 mm | 9 | 6 | 66.7% |
| Safe Zone | ≥ 2.23 mm | 39 | 37 | 100.0% |

**Table legend**. MT3: mucosal thickness assessed 3 mm apical to the mucosal margin. STM: soft tissue margin.
