# Supplementary material for: Protective factors and critical mucosal thickness for the stability of the peri‐implant soft tissue margin over 5 years: A longitudinal cohort study
Source: J Periodontol. 2026 Jul 12;97(10):2136–49. doi: 10.1002/jper.70159 (PMC13743502; doi:10.1002/jper.70159)
Supplement: Supplementary file 6 [file JPER-97-2136-s007.docx]

Supplementary Table 4. Pearson Correlation Matrix evaluating relationships between the key variables. The strongest association was a moderate positive correlation between KM width and BBT (r = 0.523, p < 0.0001), suggesting a pattern consistent with a generally thicker tissue phenotype. The other significant correlations were weak to weak-moderate, indicating that the predictors represent distinct anatomical features rather than being proxies for one another. The negative, weak-moderate correlations with BBD suggest that thinner tissue (MT1, KM width, and BBT) tend to be associated with a greater extent of BBD.

|  | MT1  r (p-value) | MT3  r, p-value | KM width  r, p-value | BBT  r, p-value | BBD  r, p-value |
| --- | --- | --- | --- | --- | --- |
| MT1 | 1.000 | 0.381(<0.001)* | 0.147 (0.092) | 0.163 (0.064) | -0.216 (0.012)* |
| MT3 | 0.381 (<0.0001)* | 1.000 | 0.262 (0.002)* | 0.186 (0.035)* | -0.153 (0.079) |
| KM width | 0.147 (0.092) | 0.262 (0.002)* | 1.000 | 0.523 (<0.0001)* | -0.369 (<0.0001)* |
| BBT | 0.163 (0.064) | 0.186 (0.035)* | 0.523 (<0.0001)* | 1.000 | -0.363 (<0.0001)* |
| BBD | -0.216 (0.012)* | -0.153 (0.079) | -0.369 (<0.0001)* | -0.363 (<0.0001)* | 1.000 |

**Table legend.** BBD: buccal bone dehiscence. BBT: buccal bone thickness. KM: keratinized mucosa. MT1: mucosal thickness assessed 1 mm apical to the soft tissue margin. MT3: mucosal thickness assessed 3 mm apical to the soft tissue margin. r: Peason correlation coefficient. *denotes statistically significance.
